# Impact of truck contamination and information sharing on foot-and-mouth disease spreading in beef cattle production systems

**DOI:** 10.1371/journal.pone.0240819

**Published:** 2020-10-16

**Authors:** Qihui Yang, Don M. Gruenbacher, Jessica L. Heier Stamm, David E. Amrine, Gary L. Brase, Scott A. DeLoach, Caterina M. Scoglio

**Affiliations:** 1 Department of Electrical and Computer Engineering, Kansas State University, Manhattan, KS, United States of America; 2 Department of Industrial and Manufacturing Systems Engineering, Kansas State University, Manhattan, KS, United States of America; 3 Beef Cattle Institute, College of Veterinary Medicine, Kansas State University, Manhattan, KS, United States of America; 4 Department of Psychological Sciences, Kansas State University, Manhattan, KS, United States of America; 5 Department of Computer Science, Kansas State University, Manhattan, KS, United States of America; The University of Hong Kong, CHINA

## Abstract

As cattle movement data in the United States are scarce due to the absence of mandatory traceability programs, previous epidemic models for U.S. cattle production systems heavily rely on contact rates estimated based on expert opinions and survey data. These models are often based on static networks and ignore the sequence of movement, possibly overestimating the epidemic sizes. In this research, we adapt and employ an agent-based model that simulates beef cattle production and transportation in southwest Kansas to analyze the between-premises transmission of a highly contagious disease, foot-and-mouth disease. First, we assess the impact of truck contamination on the disease transmission with the truck agent following an independent clean-infected-clean cycle. Second, we add an information-sharing functionality such that producers/packers can trace back and forward their trade records to inform their trade partners during outbreaks. Scenario analysis results show that including indirect contact routes between premises via truck movements can significantly increase the amplitude of disease spread, compared with equivalent scenarios that only consider animal movement. Mitigation strategies informed by information sharing can effectively mitigate epidemics, highlighting the benefit of promoting information sharing in the cattle industry. In addition, we identify salient characteristics that must be considered when designing an information-sharing strategy, including the number of days to trace back and forward in the trade records and the role of different cattle supply chain stakeholders. Sensitivity analysis results show that epidemic sizes are sensitive to variations in parameters of the contamination period for a truck or a loading/unloading area of premises, and indirect contact transmission probability and future studies can focus on a more accurate estimation of these parameters.

## 1. Introduction

Foot-and-mouth disease (FMD) is a highly contagious infectious disease that could threaten cloven-hoofed animals worldwide [1]. The FMD outbreak in 2001 in the United Kingdom resulted in severe economic losses totaling more than £2.8 billion and required the slaughter of approximately 6.5 million animals [2]. Recently, several major outbreaks have occurred in previously FMD-free countries, including South Korea and Japan [3–5]. Although the United States has been free of FMD since 1929, the disease is still prevalent in approximately two-thirds of the world [6]. Concerns about the reintroduction of FMD into the United States have escalated due to increases in international travel and trade [7]. The beef cattle industry is of great importance to the U.S. economy, and an FMD outbreak would produce devastating economic losses as estimated by simulation-based studies. For example, Pendell et al. [8] reported that an FMD virus released from the National Bio and Agro-Defense Facility in Kansas could cause $16 billion–$140 billion in damages. Schroeder et al. [9] estimated that a hypothetical FMD outbreak in the midwestern United States could result in $56 billion–$188 billion of producer and consumer losses.

The FMD virus can be transmitted between farms through animal movement (direct contact) and via fomites such as contaminated equipment and vehicles (indirect contact) [10–12]. Its transmission routes also include local area spread and airborne spread. While disease transmission through the movement of infected cattle has been extensively studied in previous works [13–15], indirect contact through fomites has recently gained attention, especially after the 2001 FMD outbreak in the United Kingdom, in which new cases occurred for several months after early implementation of an animal movement ban [16]. The spread of FMD stopped only after strict biosecurity measures targeting the movement of contaminated equipment and personnel were implemented [17]. This study focused on indirect contact via livestock transporters, one of the most at-risk operator categories [10, 18].

Only a few studies have considered truck movements to be indirect contact routes for the spread of disease among farms. Thakur et al. [19] developed a farm-level model to simulate the spread of porcine reproductive and respiratory syndrome virus through animal movement and truck sharing. Their results of using static links between farms highlighted the significant role indirect contact played in spreading the disease. Wiltshire [20] developed a model to heuristically generate a dynamic hog production system while accounting for truck contamination, demonstrating that producer specialization can increase system vulnerability to disease outbreaks. Bernini et al. [2] developed a two-layer temporal network to model disease transmission through cattle exchanges and transportation and compared epidemic size under full or partial knowledge of daily truck itineraries. Their work concluded that an accurate description of indirect contact is essential for precise prediction of epidemic spreading dynamics. However, no previous studies have included trucks as independent epidemiological units, as is emphasized in this research.

Simulation models have been widely used to mimic FMD transmission among farms and analyze various control strategies. Bate et al. [7] simulated FMD transmission in a three-county area in California, demonstrating the effectiveness of preemptive culling of highest-risk herds and ring vaccination. This model was widely used and later adapted to analyze FMD mitigation strategies in other countries, including Sweden [21] and Denmark [11]. Other stochastic, state-transition simulation models included the NAADSM [22], AusSpread [23], InterSpreadPlus [24], and AADIS [25], which involve multiple animal species and pathways for disease spreading. However, these herd-level models rely on accurate estimates of the contact frequency and distance distribution between livestock operations and do not consider actual contact sequences that occur between farms [2]. In addition, the number of animals is often assumed to be constant over time in each herd in these models.

Suggested animal movement traceability systems in the United States have faced opposition from beef producer organizations due to privacy issues, resulting in contact rates used for most FMD simulation models being based on expert opinions and questionnaires. Though the inspection at slaughterhouses is the most effective surveillance component for early epidemic detection [26], most FMD simulation models are built without including slaughterhouses. In addition, they are often based on static networks and ignore the system’s time-varying structure, which is crucial for simulating the dynamics of highly contagious diseases [27]. In this study, the structures of both cattle movement and truck movement networks vary over time based on cattle trading among farms. Recent work has focused increasingly on temporal network measures, showing that possible outbreak sizes may be overestimated in a static view of the network [28]. Sterchi et al. [29] concluded that information about transport sequences could change the contact network topology and that consideration of truck sharing and contamination could increase network connectivity and individual connectedness of farms. Liu et al. [30] built a spatially explicit cattle-level agent-based model for two counties in Kansas, in which producers made decisions on cattle trade based on cattle weights and market conditions. Their work emphasized the influence of trading dynamics on the disease transmission through cattle movement.

Traceability programs, however, have become common in the global beef market, and lack of such programs may decrease export markets for the U.S. beef industry [31]. For example, if 25% of beef products became unacceptable in international trade, then the U.S. economy would experience an estimated $6.65 billion loss [32]. An improved information infrastructure with traceability systems would yield many benefits, including targeted and timely product recalls after a foodborne illness outbreak and increased brand value for products due to quality assurance. Several pilot projects have recently developed and tested purpose-built cattle disease traceability infrastructures, such as BeefChain and CattleTrace. In these projects, cattle movement information is uploaded to a secure third-party database through radio frequency identification tags or similar devices. Since the acceptance of such traceability programs depends on the trust among stakeholders, these pilot programs have focused on persuading operators to participate. New technologies such as Blockchain, which has been increasingly studied [33–36], are expected to build trust among food partners, promote livestock traceability, and enhance food safety.

In response to potential FMD outbreaks, governmental agencies in the United States have designed control measures and preparedness plans. According to the U.S. Department of Agriculture, the depopulation of clinically affected and in-contact susceptible animals (stamping-out) is typically applied with other emergency vaccination strategies depending on the circumstances and epidemic sizes of FMD outbreaks [6]. In Kansas, for example, the Kansas Animal Health Commissioner would issue a state-wide stop movement order to all animal and related product movement if an FMD case occurred in North America. This movement restriction would remain in effect until the situation was deemed safe for the Kanas livestock industry, and producers would have to provide documents such as normal health status for animals on the production site for the previous 14 days to request a movement permit from the Kansas Department of Agriculture [37].

This work aims to *examine the potential impact of truck contamination and information sharing for FMD virus transmission in the beef cattle production system in southwest Kansas* (SW KS), *United States*. We simulate a hypothetical FMD transmission through cattle movement and truck movement with major expansions relative to the model in [38], including state transition models embedded in cattle agent, truck agent, producer agent, and packer agent. In order to enable information-sharing functionality, each producer/packer agent stores time-stamped trade information and can inform their trade partners during an outbreak. Scenario analysis is conducted to evaluate the effect of various control strategies on the spread of the epidemic, and sensitivity analysis is implemented to examine the outcomes affected by different input parameters.

This study does not mean to predict the spread of FMD in a real-world production system, but it facilitates a realistic epidemiological model to highlight the impact of indirect contact through truck movement and the potential benefits of information sharing to the system regarding disease transmission. The findings are expected to benefit existing disaster preparedness and promote the development of new mitigation strategies informed by information sharing for rapid detection and containment.

## 2. Materials and methods

In this section, we describe the agent-based model developed for the simulations and the design for scenario analysis and sensitivity analysis.

### 2.1 Model description

In this work, an agent-based, stochastic, cattle-level simulation model is developed in AnyLogic software based on a previous model. To the best of the authors’ knowledge, actual cattle and truck movement data in southwest Kansas are not accessible due to privacy issues. Based on regular business operating principles and assumed conditions obtained from the literature review, beef cattle experts, and field studies, Yang et al. designed the model in [38] and generated realistic synthetic cattle and truck movement data among premises on a daily basis. Due to the model’s large complexity, we have described mainly the major expansions to the model in [38]. Agent functionalities are altered to enable FMD transmission through both direct and indirect contact routes and information-sharing functionality.

#### 2.1.1 Model structure

**Fig 1:**
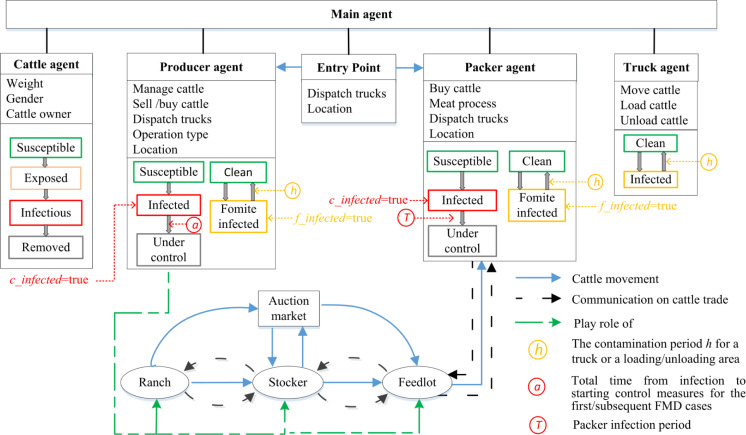
Model structure.

The model simulates the behaviors of the interdependent beef cattle production and transportation systems in SW KS, which can be regarded as dynamic cattle movement and truck movement networks. The model structure is shown in Fig 1.

As shown in Fig 1, the main agent builds the environment in which other agents live. All premises are located based on their latitude and longitude, and trucks are moving cattle on the roads within the GIS map. Producer agents play the role of cow-calf ranches, stockers, and feedlots. In ranches, cows produce a new generation of calves that are fed until around 450 pounds, and will be sold directly or via auction markets to stockers. At approximately 650 pounds, cattle raised in stocker operations will be sold directly or via auction markets to feedlots. Once heifers and steers in feedlots achieve 1250 pounds and 1350 pounds respectively, they will be moved to the final meat-processing facilities, the packers. Please note that slaughterhouses are also called packers in U.S. beef production systems, and these two terms are used interchangeably in this paper. When producers from inside SW KS request cattle from the outside, cattle coming from outside SW KS are generated at entry point agents, which are located on the major highways into and out of SW KS. Trucks are dispatched by their owner to transport cattle among premises following the amounts specified in the order agents, which are set up during the cattle trade process.

The study population and parameters used for simulation are presented in Table 1.

**Table 1:** Study population and parameters used for simulating the FMD spread.

Parameters	Value	References
Total producers (*N*)	301	Yang et al. [38]
Ranches	18 (5.98%)	
Stockers	50 (16.61%)	
Feedlots	233 (77.41%)	
Total cattle inventory at the start of the simulation	2,913,007	
Ranches	14,050	
Stockers	79,768	
Feedlots	2,819,189	
Probability of transmission when an infected cattle agent contacts a susceptible cattle agent	0.95	Boklund et al. [11]
Length of the cattle latent period (days) [Pert distribution]	Pert(1.2, 1.2, 2.4)	Yadav et al. [39]
Length of the cattle infectious period (days) [Normal distribution]	Normal(11.4, 1.1)	Yadav et al. [39]
Total time from infection to starting control measures for the first infected FMD case [Triangular distribution]	*μ* = 8.6; 6.0≤*x*≤12.8	Walz et al. [40]
Total time from infection to starting control measures for subsequent FMD cases [Triangular distribution]	*μ* = 6.6; 4.5≤*x*≤10.5	Walz et al. [40]
The contamination period *h* for a truck or a loading/unloading area of premises (days)	14	Rossi et al. [41]
Length of the packer infection period (days) [triangular distribution]	*μ* = 5; 0≤*x*≤10	Wiltshire et al. [20]
Probability that truck will be contaminated upon visiting an infected producer/packer	0.15	Wiltshire et al. [20]
Probability that contaminated truck will infect the subsequent producer/packer it will visit	0.15	Wiltshire et al. [20]
Probability that infected cattle will contaminate packer/producer receiving area	0.75	Wiltshire et al. [20]
Number of days to trace back and forward during the information sharing process (days)	14	Assumed

#### 2.1.2 Epidemic initialization

All the cattle in the system are in the susceptible state before the epidemic is initialized. For each simulation run, one randomly selected animal from outside SW KS will enter the infectious state on the 9^th^ day midnight, and will be brought to a stocker inside the region on the 10^th^ day. If there are no cattle at the border at this time, the simulation will stop and start the next simulation run. In different simulation runs, the recipient stocker of the first infectious animal will be different due to the stochasticity of the model.

#### 2.1.3 Spread of the disease

In this section, we describe major functions added to the base model to enable FMD transmission.

(1) Cattle agent

Each cattle agent is associated with a Susceptible-Exposed-Infectious-Removed compartment model. Compared to the cattle in the infectious state, cattle agents in the exposed state have been infected by the FMD disease, but are not contagious yet. Animals in the removed state have been infected before, and become dead or immune, or are culled by the producers. More specifically, in contact with an animal in the infectious state, a susceptible cattle agent has a 95% chance to become infected and transition to the exposed state. After a latent period, the cattle agent will transition to the infectious state during which it contacts and infects other cattle of the same premises. Then, the cattle agent enters the removed state after an infectious period.

Considering computational efficiency, we use a scaling factor of 10 to change all the parameters related to the number of cattle, e.g., truck capacity and cattle capacity of each producer, such that one cattle agent represents ten cattle. We assume that each cattle agent contacts, on average, 20 cattle agents in a day. 20 cattle agents correspond to 200 cattle due to the scaling factor, indicating the approximate number of cattle in a group. Accordingly, during the infectious state, each cattle agent can infect on average 20*0.95 cattle agents per day, where 0.95 is the probability of transmission when infected cattle contact susceptible cattle.

(2) Producer agent

When an infectious cattle agent occurs in the producer agent, the producer will transition from the susceptible state to the infected state (*c_infected* = true). If there is no control strategy implemented on the producer premises level, the infected producers will not transition to the under-control state. Otherwise, the producer agent will enter the under-control state after a total time from infection to starting control measures. As the producer agent enters the under-control state, all its cattle will transition to the removed state, simulating that the producer depopulates its cattle. In addition, various control strategies are implemented, and more details will be described in the scenario analysis section.

When all infected cattle of an infected producer enter the removed state, the *c_infected* variable is set to be false. On the other hand, when a producer becomes infected by fomite, the variable *f_infected* becomes true and will last for 14 days (the contamination period *h* in Table 1).

(3) Packer agent

Once a cattle agent in the infectious state arrives at the packer, the packer agent will transition to the infected state (c*_infected* = true), and after a packer infection period *T*, the packer goes to the under-control state, in which the packer stops requesting and transporting cattle from other producers to its location. To be more realistic, we assume that more infectious cattle arriving at the packer will speed up FMD detection. For example, if there are infectious cattle coming in on day *d*_1_ and day *d*_2_, the model will generate two numbers, *ct* and *ct*’, respectively, according to the distribution with a mean of 5 days in Table 1. The smaller value between *ct*’ and *ct* will be assigned as the contamination period *T*, as shown in Fig 1. On the other hand, once the packer is infected by fomite, its cattle receiving area will remain contaminated (*f_infected* = true) for 14 days based on the contamination period *h*.

(4) Truck agent

Following a clean-infected-clean cycle, trucks move to the origin premises to pick up cattle and then move the animals to the destination premises to unload. With direct contact infection probability set as 1.0 [19, 41], when at least one infectious cattle agent is moved and received at the destination premises, the destination becomes infected with *c_infected* set as true. For indirect contact, trucks may become contaminated by visiting fomite-infected premises, and then remain contaminated for a contamination period *h*, during which time the infection may spread to other premises.

More specifically, when the truck arrives at the origin premises, if there are infectious cattle loaded to the truck at the origin premises, then there is a 75% probability that the receiving area of the origin producer will become infected via fomite (*f_infected* = true). If the origin producer is fomite-infected and the truck is not contaminated, the truck may become contaminated with a 15% probability; however, if the truck is contaminated and the origin producer is not fomite-infected (*f_infected* = false), the truck will cause the producer to become fomite-infected with a 15% probability.

When the truck arrives at the destination premises, if there are infectious cattle unloaded, there is a 75% probability that the destination premises will become fomite-infected. Meanwhile, if the destination is fomite-infected and the truck is clean, there is a 15% probability that the truck may become contaminated. However, if the truck is infected and the destination is not fomite-infected, the truck may cause the destination to become fomite-infected. Note that at the time the origin or destination producer becomes fomite-infected, we will randomly select one cattle agent from the premises to become infected.

### 2.2 Scenario analysis

In this section, ten different scenarios are constructed based on three attributes: (1) the two FMD virus transmission routes, namely by direct contact only or by both direct and indirect contact; (2) the implementation of movement bans; and (3) the involvement of information infrastructure. The combination of these factors is described in Table 2, and all the parameters follow the values described in Table 1 in the scenario analysis. Five hundred iterations of each scenario are run to generate a distribution of the outcomes. A 200-day simulation duration is selected such that all producers already reach the steady-state in the end regarding disease transmission, i.e., no new infections occur.

**Table 2:** Description of the scenarios.

Scenario number	Scenario name	Spread by direct contact	Spread by indirect contact	Movement ban on infected producers	Information infrastructure enabled on producers	Information infrastructure enabled on packers	Movement ban in the whole region
1	DI	Yes	No	No	No	No	No
2	DI_B	Yes	No	Yes	No	No	No
3	DI_B_P	Yes	No	Yes	Yes	No	No
4	DI_B_PP	Yes	No	Yes	Yes	Yes	No
5	DI_RB	Yes	No	Yes	No	No	Yes
6	D&IN	Yes	Yes	No	No	No	No
7	D&IN_B	Yes	Yes	Yes	No	No	No
8	D&IN_B_P	Yes	Yes	Yes	Yes	No	No
9	D&IN_B_PP	Yes	Yes	Yes	Yes	Yes	No
10	D&IN_RB	Yes	Yes	Yes	No	No	Yes

DI: direct contact only, D&IN: direct and indirect contact, B: movement ban on infected producers, P: information infrastructure on producers, PP: information infrastructure on both producers and packers. RB: region-wide movement ban.

In scenarios 1 and 6, there is no control strategy implemented on the producer premises level. In all the other scenarios, once a producer/packer is in the under-control state, it will be under movement restrictions. Here we assume that any movement ban was effective with a 100% compliance, meaning that all movements to and from the producer/packer would stop, once the movement ban is enacted. Therefore, both direct and indirect contacts related to these premises are stopped, since trucks will no longer move cattle to or from these premises. To enable information-sharing functionality, each producer/packer agent stores time-stamped trade information with its trade partners. During outbreaks, the producer/packer agent can trace back and forward its trade record in the past 14 days to inform their trade partners about its infection by disease. This time-interval of 14 days will be referred as the number of days to trace back and forward in the sensitivity analysis section.

More specifically, (i) for scenarios with a movement ban on infected producers, or called producer isolation (scenarios 2 and 7), a movement ban will be introduced to the infected producer once the producer is in the under-control state. (ii) For scenarios with a region-wide movement ban (scenarios 5 and 10), the movement ban will be enacted for all the producers and packers inside the region as long as there is one infected producer in the under-control state. (iii) For scenarios with information-sharing functionality enabled only on producers and not on packers (scenarios 3 and 8), the producer in the under-control state will notify those producers with which it has traded in the past 14 days (both its suppliers and customers). Once those producers receive notification from the information sender, they will go to the under-control state and notify their trading partners as well. (iv) For scenarios that information-sharing functionality is enabled on both producers and packers (scenarios 4 and 9), the producer will notify both producers and packers with which it has traded in the past 14 days. In addition, once an infected packer enters the under-control state, it will immediately notify its trade partner producers. On the other hand, if a producer in the under-control state notifies a packer not in the under-control state that it has the potential to be infected, the packer will also go to the under-control state.

### 2.3 Sensitivity analysis

To evaluate how changes in parameters can impact the simulation results, we perform sensitivity analyses for different scenarios by creating variations in a single input parameter while keeping other simulation settings unchanged. First, sensitivity analyses of the number of days to trace back and forward during the information-sharing process are conducted under scenarios D&IN_B_PP (information infrastructure enabled on producers and packers) and D&IN_B_P (information infrastructure enabled on only producers). The number of days to trace back and forward is selected for sensitivity analysis, as it is assumed as 14 days based on expert opinion, and it is likely to influence the epidemic control effectiveness during the information-sharing process. Second, parameters related to indirect contact routes: (a) indirect contact transmission probability, and (b) contamination period *h* for a truck or a loading/unloading area of premises are altered, and their impact on the number of infected producers are evaluated under scenario D&IN_B. In this work, we set indirect contact transmission probability as 0.15 based on [20], but Bate et al. [7] assume distributions for the high-risk and low-risk indirect contact probability as BetaPert(0.1,0.5,0.9) and BetaPert(0.05,0.175,0.35), respectively. Accordingly, we compare the number of infected producers under different values of the indirect contact transmission probability, varying from 0.05 to 0.5 with an interval of 0.05. In addition, we conduct sensitivity analysis on the contamination period *h*, ranging from 1 day to 22 days with an interval of 3 days. For all variations, 500 iterations of each scenario are simulated for 200 days.

## 3. Results

### 3.1 Scenario analysis

**Fig 2:**
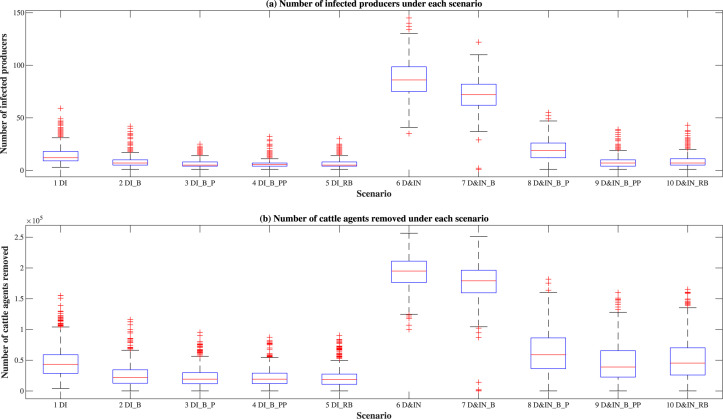
Distributions of numbers of infected producers and cattle agents removed.

Fig 2 shows the distributions of numbers of infected producers and cattle agents removed for the ten scenarios. Overall, the epidemic size is smaller for scenarios that model only direct contact (scenarios 1–5) when compared to equivalent scenarios that incorporate both direct and indirect contact (scenarios 6–10). For example, the median number of infected producers changes from 12 in scenario 1 (direct contact only) to 86 in scenario 6 (direct and indirect contact). Findings indicate that including indirect contact through truck contamination has a considerable impact on the FMD spread among producers. Since contaminated trucks can travel to multiple places, truck contamination can aggravate the disease spread between those who do not have direct contact (animal movement in between), thereby enlarging the scale of epidemic spreading.

More specifically, for scenarios 1–5, scenarios 2–5 result in a smaller and similar epidemic size compared to scenario 1 (no premises-level control strategies implemented). For scenarios 6–10, scenario 6 (no premises-level control strategies implemented) results in the largest epidemic size, followed by scenario 7 (producer isolation), and then scenario 8 (information infrastructure enabled on producers), while scenarios 9 (information infrastructure enabled on producers and packers) and 10 (regional movement ban) result in the smallest and similar epidemic sizes. There is a slight difference in terms of the median size between scenarios 6 and 7, indicating that merely implementing movement bans on infected producers cannot effectively contain the epidemic when indirect contact is considered. Information sharing can significantly reduce the epidemic size, compared to scenario 6. Particularly, the number of infected producers is larger in scenario 8 with median 19 and interquartile range 12–26, compared with scenarios 9 and 10 in Fig 2A. This is because information-sharing functionality is not enabled on packers in scenario 8, and these infected packers can spread the infection by their contaminated trucks to other producers in some simulation runs.

**Fig 3:**
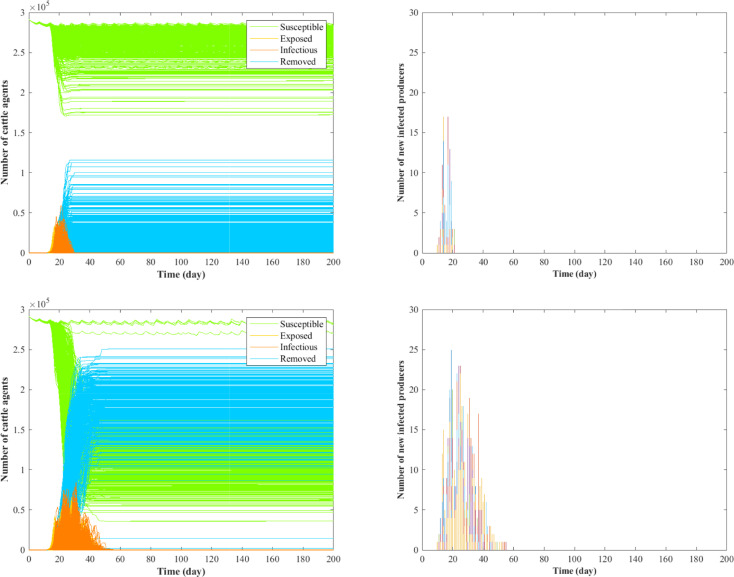
Comparison between scenarios 2 (top) and 7 (bottom). In the figure, various colors represent the number of new infected producers in different runs.

**Fig 4:**
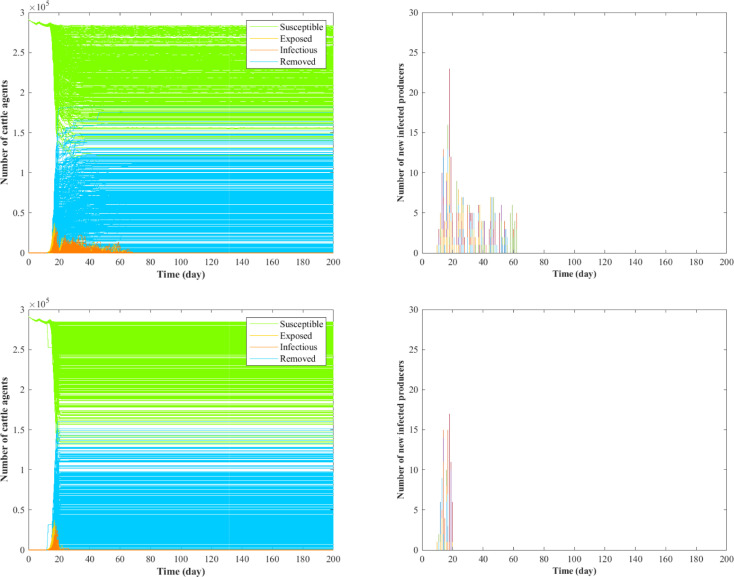
Comparison between scenarios 8 (top) and 9 (bottom). In the figure, various colors represent the number of new infected producers in different runs.

As additional information for the epidemic dynamics, Figs 3 and 4 show the numbers of cattle agents in each compartment and newly infected producers over time for scenarios 2, 7 and 8–9. In Fig 3, both the epidemic size and epidemic duration is larger in scenario 7 compared with scenario 2, indicating that truck contamination can aggravate the spread of epidemics. Comparing Fig 4 with Fig 3, it is shown that the addition of information-sharing functionality reduces the number of cattle agents removed during the outbreak. Particularly, there is a larger variance in terms of new infected producers in scenario 8 compared to scenario 9, indicating that the system is more robust when information-sharing functionality is enabled in both producers and packers.

**Fig 5:**
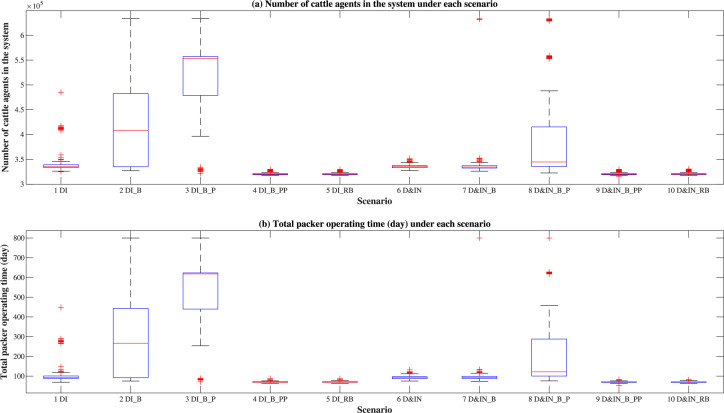
Distributions of the total number of cattle agents and total packer operating time.

In the cattle supply chain, cattle flows are essentially driven by the normal operation of packers to meet steady demand, so the total number of cattle in the system during the simulation period is strongly affected by the packers’ operation time. With infected cattle coming in, the packer will go to the under-control state after an infection period, and then will stop receiving cattle from feedlots both inside and outside the region. As a result, feedlots will stop requesting cattle from other premises, which will impact the number of cattle stocker operations will request. Therefore, the system’s cattle flow quickly stops once packers are in the under-control state, affecting the total number of cattle agents in the system. As the producers and packers gain revenues by marketing their cattle or cattle-related products, the total number of cattle agents in the system, which include the cattle that are marketed and being prepared to be marketed, can be seen as a measure for the economic impact of the control strategies on the cattle industry. In each simulation run, we calculate the total packer operating time by summing the four packers’ operating time during the 200-day simulation period. Distributions of the total number of cattle agents that exist in the system and total packer operating time are shown in Fig 5.

In Fig 5, scenarios 3 results in the largest median number of cattle agents (Fig 5A) and packer operating time (Fig 5B), followed by scenario 2 (producer isolation), and all the other scenarios are with a similar value. In scenario 3 (information-sharing functionality enabled only on producers), the infected producers and their trading partner producers become isolated in the system quickly, but the packers can continue to request cattle from other uninfected producers, thereby sustaining regular operations for a longer time. Compared with scenario 1 (no farm-level control strategies implemented), the median number of cattle agents of scenario 2 is larger because producer isolation is already effective to contain the epidemic, resulting in less infected packers and an increased packer operation time. However, the infected producers will be in the under-control state more quickly in scenario 3 with information-sharing among producers, so packers can sustain regular operations for a longer time, and the median number of cattle agents in scenario 3 is higher compared to scenario 2.

When information sharing is enabled for both producers and packers (scenarios 4 and 9), once infected feedlots occur, the four packers are much likely to have traded with these producers and will quickly transition to the under-control state, resulting in a lower number of cattle agents over the system. Though information-sharing on all premises and region-wide movement ban (scenarios 4–5, 9–10) are both very effective regarding the epidemic containment, these two relatively protective strategies have a greater economic impact with a higher loss in cattle. It indicates that determining the appropriate control strategy is a tradeoff between multiple factors, including the economic effect and effectiveness of the epidemic control. Based on these simulations, placing control measures on packers has a large impact on the business discontinuity to the system based on decreased packer operating time and number of cattle agents. These control decisions need to be based on additional criteria not considered in the current setting, where a packer comes under control only because one of its trading partners is under control.

### 3.2 Sensitivity analysis

#### 3.2.1 The number of days to trace back and forward during the information-sharing process

**Fig 6:**
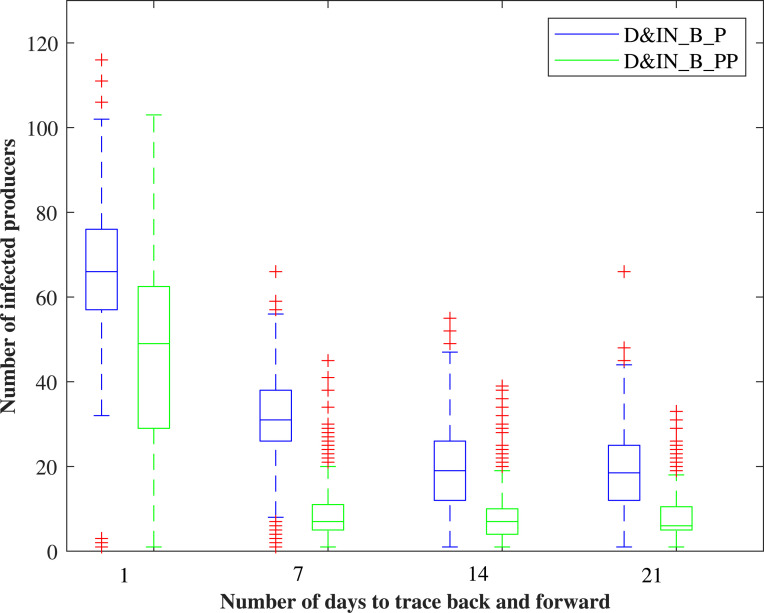
The sensitivity of the number of days to trace back and forward during information sharing.

The distribution of the number of infected producers under scenarios D&IN_B_P (information infrastructure enabled on producers) and D&IN_B_PP (information infrastructure enabled on producers and packers) is shown in Fig 6. The modeled outcomes are sensitive to the variation in the number of days to trace back and forward. For instance, a decrease from 14 days to 1 day in scenario D&IN_B_P (scenario 8) results in an increase of 2.47 times in the median number of infected producers. A small number of days to trace back and forward is not enough to contain the epidemic, but a larger value can lead to more premises in the under-control state, resulting in a larger economic impact. This implies that the number of days to trace back and forward is an important parameter during the information-sharing process and should be paid much attention in practice. Besides, the variation regarding the epidemic size in scenario D&IN_B_PP (scenario 9) is smaller than scenario D&IN_B_P, indicating that it is necessary to include the critical component packers into the information-sharing infrastructure.

#### 3.2.2 Indirect transmission probability and contamination period *h*

**Fig 7:**
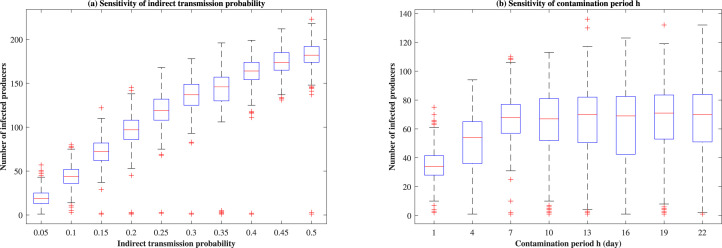
The sensitivity of indirect transmission probability and contamination period *h* under scenario D&IN_B.

The indirect transmission probability used in the scenario analysis equals 0.15, referring to the probability that a truck will become contaminated upon visiting an infected producer/packer, and the probability that contaminated truck will infect the subsequent producer/packer. Results in Fig 7 are both sensitive to the indirect contact transmission probability and the contamination period *h* for scenario D&IN_B (producer isolation). For example, an increase of indirect transmission probability from 0.15 to 0.2, the median number of infected producers changes from 72 to 97.

The sensitivity in outcomes to changes in indirect contact transmission probability (Fig 7A) and the contamination period *h* (Fig 7B) suggests that a more accurate estimation of these parameters related to indirect contact routes are critical to epidemic modeling. Since these two parameters are closely tied to the biosecurity status of cattle premises, these premises should be vigilant and follow guidelines for proper cleaning and disinfection of vehicles and cattle loading/unloading areas, such to limit the impact of indirect contacts on disease spread between premises.

## 4. Discussion and conclusion

This study aims at examining the potential impact of truck contamination and information sharing on epidemic dynamics, with a particular focus on indirect contacts due to cattle movements. Based on the agent-based model developed in [38], we design a between-farm FMD virus spread model by explicitly simulating the beef cattle production and transportation in southwest Kansas. This model makes several advances over previous epidemic models on the spread of FMD virus. Many epidemiological studies consider only direct contacts [14, 27]. Existing models such as the North American Animal Disease Spread Model [22] and InterSpread Plus [24] include the indirect contact transmission route, but do not consider the sequence of contacts occurring between farms. In this work, cattle are transported by trucks based on premises locations and regular business operating principles, which can be regarded as temporal cattle and truck movement networks. More specifically, the number of connections between pairs of farms and the strength of each connection varies over time, as each transaction between farms involves various cattle order volumes and each truck can transport a variable number of animals. What’s more, these models take each farm as an epidemiological unit and ignore the modeling of disease spread within a farm. In contrast, in our model, each cattle agent follows its Susceptible-Exposed-Infectious-Removed compartment and is with heterogeneous parameters such as weight and gender. Australian Animal Disease model [25] predicts the fraction of animals in each state of each herd, but the number of animals in each herd is constant over time, whereas ours changes according to the cattle trade information. In addition, these FMD simulation models only include producers, whereas our work includes also packers, which are sinks for cattle transported through the production system and behave as hubs that can facilitate disease spreading. Natale et al. [42] simulate a hypothetical outbreak in dairy production systems, including slaughter plants, but they only include direct contact through cattle movement, whereas ours simulates indirect contact routes resulting from loading/unloading areas of premises, and trucks transporting cattle.

Our simulation results reveal that including the truck movement (indirect contact) can significantly exacerbating the disease spread in the system, compared with equivalent scenarios that only consider animal movement (direct contact). For example, the median [interquartile range] number of infected producers is 7 [5–10] vs. 72 [62–82] in scenarios DI_B (direct contact only) and D&IN_B (direct and indirect contact) respectively. This finding is consistent with recent studies [19, 41, 43, 44], which all highlighted the substantial effect of indirect contacts on the ability of farms to potentially spread diseases. Focusing on dairy farms [45], applied network analysis techniques and showed that indirect contacts through on-farm visits by veterinarians produced a more connected network compared to direct contact only. Considering the sharing of vehicles for shipment of swine [19], concluded that indirect contact significantly played a role in further spreading the infection, which is not directly connected regardless of the types of network structures.

The duration a truck or a loading/unloading area of premises remaining contaminated, i.e., the contamination period *h*, is affected by not only the ability of the pathogen to survive in fomites, but also by environmental factors such as temperature, and by the frequency of the disinfection operations [41]. In this study, we assume the contamination period a constant number of days, i.e., 14 days, similar to the work in [2, 41, 43]. The sensitivity analyses on the contamination period and indirect contact transmission probability show their significant influence on the epidemic size. This highlights the need for a deeper understanding of indirect transmission mediated by fomites, and improved analyses or experiments need to be conducted to more accurately quantify the contamination period and indirect contact transmission probability.

Over the past decades, many studies have focused on the usage of information sharing as a method to improve supply chain performance and resilience to disruptions [46, 47]. The information shared, such as demand and inventory level, can foster the operation control and decision making about supply chain disruptions. To enhance food security by sharing the authentic data while maintaining privacy, several recent studies have analyzed food supply chain traceability using the blockchain technology [33, 34, 48, 49], which is promising to result in more information sharing.

The beef cattle production system is essentially a food supply chain, but cattle producers are reluctant to share their information due to privacy concerns. Different from prior works, the findings provided in this study add new insights to the works related to the supply chain with a focus on sharing information during epidemics. Our simulation results showed that including information-sharing functionality on producers and packers can dramatically reduce the epidemic size; for instance, the median number of infected producers in scenario D&IN_B (producer isolation) reduces by 90.28% in scenario D&IN_B_PP (information infrastructure enabled on producers and packers). Policymakers may focus more on promoting the development of new mitigation strategies informed by information sharing based on novel cyber-infrastructure for rapid detection and containment. Particularly, the number of days to trace back and forward during information sharing has a significant influence on the simulation outcome and is worth further studying considering the economic impacts of the related mitigation strategies. These findings supported previous theoretical studies that showed the effectiveness of information awareness or information diffusion in suppressing disease spreading using multilayer networks [50–53]. In these studies, individuals become alerted and adopt preventive measures when sensing the infection in their neighbors.

Finally, it should be noted that we include only truck movement and ignore other forms of indirect contact, such as the movement of personnel or equipment. In this study, we compared the disease spread under the scenario of direct contact only and scenario of indirect and direct contact. It would also be interesting to model a scenario of indirect contact only to investigate the role of indirect contact in the disease spreading thoroughly. As the truck movement occurs for the shipment of cattle, indirect contact is always associated with direct contact. Therefore, we did not model the scenario of indirect contact only. Nonetheless, the general structure of our model makes it potentially extendible to include other potential indirect transmission routes such as feed transporters, and this can be the topic of future analyses.

Another limitation of the model concerns the underlying synthetic cattle and truck movement network, on which our analysis was based. As aforementioned, actual movement data are unavailable in southwest Kansas due to privacy concerns. The availability of high-quality actual cattle and truck movement data in the future may result in different outcomes and lead to a better understanding of the role of truck contamination in the disease spreading.

In the current model, producers/packers randomly select other producers to trade cattle, and future work can analyze the disease spread under various contact network structures by altering the current trade pattern. For simplicity, we assume that once packers are in the under-control state, they stop requesting cattle and do not go back to normal operations during the epidemic period, while this may not be what happens in practice. Future work may impart greater realism to the packer agent and further analyze the economic impact caused by different control strategies.

Another area of future work is to add an economic and social component in the agent-based model and investigate its impact on the acceptance of information sharing in the face of epidemiological threats. Whereas the model used here assumes that all producers participate in the information-sharing process.

## Supporting information

S1 Data (ZIP)
